# Who, where, when: Colorectal cancer disparities by race and ethnicity, subsite, and stage

**DOI:** 10.1002/cam4.6105

**Published:** 2023-05-22

**Authors:** Kristin M. Primm, Andrea Joyce Malabay, Taylor Curry, Shine Chang

**Affiliations:** ^1^ Department of Epidemiology The University of Texas MD Anderson Cancer Center Houston Texas USA; ^2^ School of Public Health Texas A&M University College Station Texas USA

**Keywords:** colorectal cancer, racial/ethnic disparities, SEER, stage at diagnosis, subsite, time trends

## Abstract

**Background:**

There are well‐established disparities in colorectal cancer (CRC) outcomes between White and Black patients; however, assessments of CRC disparities for other racial/ethnic groups are limited.

**Methods:**

The Surveillance, Epidemiology, and End Results database identified patients aged 50–74 years with CRC adenocarcinoma from 2000 to 2019. Trends in age‐adjusted incidence rates were computed by stage at diagnosis and subsite across five broad race/ethnic groups (White, Black, Asian/Pacific Islander [API], American Indian/Alaskan Native [AIAN], and Hispanic) and four API subgroups (East Asian, Southeast Asian, South Asian, and Pacific Islander) Multivariable logistic regression evaluated associations between race/ethnicity and diagnosis stage. Multivariable Cox proportional hazards models assessed differences in cause‐specific survival (CSS).

**Results:**

Hispanic, AIAN, Southeast Asian, Pacific Islander, and Black patients were 3% to 28% more likely than Whites to be diagnosed with distant stage CRC, whereas East Asian and South Asians had similar or lower risk of distant stage CRC. From Cox regression analysis, Black, AIAN, and Pacific Islanders also experienced worse CSS, while East Asian and South Asian patient groups experienced better CSS. No significant differences in CSS were observed among Hispanic, Southeast Asian, and White patients. When stratified by stage, Black patients had worse CSS across all stages (early, hazard ratio (HR) = 1.38; regional, HR = 1.22; distant, HR: 1.07, *p* < 0.05 for all).

**Conclusion:**

Despite advances in CRC screening, treatment and early detection efforts, marked racial/ethnic disparities in incidence, stage at diagnosis, and survival persist. Findings demonstrate the extent to which aggregating heterogenous populations masks significant variability in CRC outcomes within race/ethnic subgroups.

## 1 INTRODUCTION

Colorectal cancer (CRC) incidence rates (IRs) have declined in recent decades following the adoption of national screening programs for adults aged between 50 and 75 years.^1^ Despite this progress, CRC outcomes vary considerably by race/ethnicity. CRC disproportionately affects Black Americans, who experience higher CRC incidence and mortality rates and lower survival rates compared to other racial/ethnic groups.^1^, ^2^, ^3^, ^4^, ^5^, ^6^ While Asians and/or Pacific Islanders (API) as a whole experience lower CRC incidence and better survival than other race/ethnic groups, substantial variation in cancer outcomes within district API subgroups have been reported.^7^, ^8^, ^9^

Multiple studies reporting progress over time in CRC including by race/ethnicity imply amelioration of critical cancer disparities.^3^, ^4^ While heartening declines in trends resulting in part from concerted efforts to reduce CRC in the overall population have been reported, some overlooked disparities may persist due to limitations in study design and generalizability of findings. For example, studies of racial disparities in cancer outcomes are frequently limited to Black and White comparisons^2^, ^3^, ^6^, ^10^ and recent assessments of CRC disparities for other patient populations (e.g., disaggregated subgroups of API) are lacking. Furthermore, few recent studies have examined the impact of both stage at diagnosis and anatomical subsite on CRC disparities by race/ethnicity^11^, ^12^ and it remains unclear whether stage of CRC diagnosis and anatomical subsite together have important impact on disparities nor whether these trends vary significantly by racial and ethnic group.

Given widely reported variability by racial/ethnic group in the engagement with population‐level early detection CRC screening,^2^ this analysis evaluates the influence of both stage at diagnosis and anatomical site on CRC disparities to observe whether all racial/ethnic groups have benefitted equally. Using data from the Surveillance, Epidemiology, and End Results Program^13^ (SEER), we examined trends and disparities in CRC incidence and survival by race/ethnicity, subsite, and stage at diagnosis among individuals diagnosed with colorectal adenocarcinoma from 2000 to 2019. We limit our analysis to the most commonly diagnosed CRC histology (i.e., adenocarcinoma) and patients between ages 50–74 years, a histological subtype and age group that reflects the target of most CRC screening and prevention programs during the period of analysis.

## 2 METHODS

The data were obtained from the National Cancer Institute's SEER 17 database (November 2021 submission).^13^ The SEER program collects information on patient demographics, primary tumor site, histology, stage at diagnosis, and course of treatment from population‐based cancer registries covering approximately 27 percent of the U.S. population. All SEER data were publicly available, de‐identified, and therefore, determined to be exempt from Institutional Review Board review.

The study population included adult patients aged 50–74 years diagnosed CRC from 2000 to 2019. CRC cases were identified based on International Classification of Diseases for Oncology, third revision (ICD‐O‐3) codes and stratified by tumor subsite (proximal colon, ICD‐O‐3 codes C18.0, C18.2‐ C18.4; distal colon, C18.5‐C18.7; rectum, C19.9, C20.9). Overall CRC included these three anatomic locations in addition to “large intestine not otherwise specified” (C26.0). We excluded tumors originating in the appendix, as they are considered distinct from those arising in the colorectum.^14^ Cases were limited to adenocarcinoma, the most common CRC histology.

Race/ethnicity information in the SEER dataset was collected from medical records and first categorized into five broad groups: Non‐Hispanic (NH) White, NH Black, NH American Indian/Alaskan Native (AIAN), NH API (referred to hereafter as White, Black, AIAN, and API), and Hispanic. Patients with unknown or missing race or ethnicity information were excluded from the analysis (*n* = 2189). Additional subgroup analyses of API patients were conducted for the following five subgroups: East Asian (Chinese, Japanese, Korean), South Asian (Asian Indian or Pakistani), Southeast Asian (Filipino, Vietnamese, Thai, Loatian, Kampuchean, Hmong), Pacific Islander (Hawaiian, Samoan, Pacific Islander, Tongan, Fiji Islander, Guamanian, Micronesian, Polynesian, Melanesian, Chamorran, Tahitian, and New Guinean). Patients listed as “other Asian” were included in the aggregate API group but were not included in subgroup analysis (*n* = 3217). Race/ethnicity was used as a proxy measure for experiences of structural and systemic racism, resulting in the inequitable distribution of resources that cause adverse health outcomes among minority groups. Additional demographic variables included age (50–54, 55–59, 60–64, 65–69, or 70–74 years), sex, marital status at diagnosis (married [including common law marriages], widowed, separated/divorced, unmarried or domestic partner, and unknown), and county‐level median household income (<$55,000, $55,000–$64,999, $65,000–$74,999, ≥$75,000, unknown).

Stage at diagnosis was categorized as early (in‐situ or localized), regional, distant, and unknown stage using SEER summary stage definitions. Cause‐specific survival (CSS) was derived from SEER's cause‐specific death classification and vital status. Follow‐up time was measured from the date of diagnosis until death from CRC or end of follow‐up (December 31, 2019). Patients who were alive or dead due to other causes at the time of the last follow‐up were censored. Cases reported from death certificate or autopsy only, and cases with zero or missing survival time were excluded from survival analyses (*N* = 11,322).

### 2.1 Statistical analysis

We used two SEER databases to derive IR estimates for this analysis. Annual CRC IRs for White, Black, Hispanic, AIAN, and aggregate API patients were calculated from the SEER 17 incidence dataset.^13^ Because incidence data for specific API subgroups are not available in the standard SEER 17 incidence dataset, the specialized SEER 9 incidence database for detailed API groups was used to calculate age‐adjusted IRs for API subgroups.^15^

CRC IRs were calculated and stratified by stage and tumor subsite and age‐adjusted to the 2000 U.S. population using SEER*Stat software (version 8.4.0).^13^ Age‐adjusted IRs are presented by time period (5‐year groupings) with corresponding incidence rate ratios (IRRs) for comparisons by race/ethnicity (using White patients as the reference group). Joinpoint regression analysis software (version 4.9.0.1) was used to calculate annual percentage change and its *p* value. Trends and IRs based on fewer than five cases in any of the data years were considered statistically unreliable and were suppressed.

To determine whether proportions of cancer stage are shifting over time, cancer stage proportions (i.e., percent contribution of each cancer stage to overall incidence) are presented by time period (5‐year groupings) and by race/ethnicity. Chi‐squared tests for trend were used to assess trends in stage distribution in the consecutive time periods.

Univariate and multivariable logistic regression models were used to assess the association of race/ethnicity and distant stage diagnosis. The multivariable logistic regression model included adjustments for age at diagnosis, sex, marital status at diagnosis, county‐level median household income, tumor subsite, and diagnosis year. Unadjusted Kaplan–Meier survival curves with log‐rank tests assessed differences in CSS by race/ethnicity. Multivariable Cox proportional hazards regression models were used to calculate adjusted hazard ratios (HRs) and 95% confidence intervals (CIs) to assess the effect of race/ethnicity on CSS. Multivariable Cox proportional hazards models included all covariates described above and additionally adjusted for treatment via surgery (yes, no/unknown), and diagnosis stage. Separate Cox proportional hazards models were also conducted by stage at diagnosis. Additional details of the methods can be found in Supplementary Information. Proportional stage analysis and all regression analyses were performed using STATA 15 (StataCorp).^16^ All statistical tests were two‐sided with a significance level of <0.05.

## 3 RESULTS

Within the SEER 17 database, there were 371,227 cases of CRC diagnosed between 2000 and 2019 among patients aged 50–74 years, including 250,913 (67.6%) White, 45,435 (12.2%) Black, 41,314 (11.1%) Hispanic, 2804 (0.8%) AIAN, and 30,761 (8.3%) API patients (Table 1). API subgroups comprised of 13,525 (3.6%) East Asian, 10,197 (2.8%) Southeast Asian, 1751 (0.5%) South Asian, 3217 (0.9%) Other Asian, and 2071 (0.6%) Pacific Islander patients. For White, Black, Hispanic, and AIAN patients, the majority (35.6%–47.1%) of CRC tumors were located in the proximal colon. Within API subgroups, CRC tumors were most frequently located in the distal colon (East Asian, Southeast Asian, and Other Asian) or the rectum (South Asian and Pacific Islander). White patients had the highest proportions of early stage tumors (44.4%), whereas Black and Pacific Islander patients had the highest proportions of distant stage tumors (23.2% and 22.9%, respectively).

**TABLE 1:** Characteristics of colorectal cancer cases by race/ethnicity, SEER 17 2000–2019.

Characteristic	White	Black	Hispanic	American Indian	API	East Asian	Southeast Asian	South Asian	Pacific Islander
Number of cases (*N*)	250,913	45,435	41,314	2804	30,761	13,525	10,197	1751	2071
Sex									
Female	42.1	46.5	42.4	46.0	44.2	44.8	44.3	38.8	41.2
Male	57.9	53.5	57.6	54.0	55.8	55.2	55.7	61.2	58.8
Age									
50–54	14.2	17.3	18.8	16.8	17.0	15.3	17.5	17.7	19.2
55–59	16.8	19.5	19.9	19.4	18.6	17.4	18.9	19.0	21.9
60–64	20.2	21.5	20.7	22.9	20.2	19.3	21.4	20.2	20.7
65–69	23.7	22.2	21.4	20.1	22.9	24.1	22.4	24.6	20.3
70–74	25.1	19.5	19.2	20.8	21.2	23.9	19.9	18.6	17.9
Subsite									
Proximal	38.6	47.1	35.6	37.2	29.1	31.3	26.4	29.1	29.7
Distal	27.4	26.8	29.0	28.6	35.1	34.4	36.3	31.9	33.3
Rectum	31.6	22.4	32.7	31.6	34.0	32.6	35.2	37.1	34.8
Colon, NOS	2.4	3.6	2.7	2.5	2.5	1.7	2.1	1.8	2.3
Stage									
Early^a^	44.4	40.8	40.7	41.0	41.8	41.9	40.2	40.9	39.8
Regional	35.0	33.2	36.5	35.8	37.4	38.4	37.6	39.1	34.8
Distant	18.3	23.2	19.6	21.0	17.9	17.1	19.4	16.5	22.9
Unknown	2.3	2.8	3.2	2.3	2.9	2.7	2.8	3.5	2.5
Median income									
≥$75,000	29.7	18.5	24.5	43.9	53.4	55.1	50.6	52.6	60.7
$65,000–$74,999	22.8	20.1	27.1	13.1	20.5	18.9	23.1	19.3	16.0
$55,000–$64,999	21.6	26.5	32.6	13.6	22.6	24.3	22.2	20.7	20.1
<$55,000	25.9	34.9	15.8	29.3	3.5	1.7	4.0	7.4	3.2
Marital status									
Married	60.8	40.7	56.3	45.8	67.5	68.6	67.8	76.4	55.3
Divorced/separated	11.9	15.0	12.1	12.1	7.5	7.0	7.6	4.2	12.4
Single	13.1	27.7	18.1	17.1	12.2	12.4	11.9	6.3	16.9
Unmarried/domestic partner	0.2	0.1	0.2	0.5	0.1	0.1	0.1	0.1	0.2
Widowed	8.4	10.0	7.1	9.0	6.9	6.5	7.5	6.5	8.5
Unknown	5.6	6.5	6.2	15.6	5.9	5.4	5.1	6.6	6.8

*Note*: Numbers represent column percentages unless otherwise specified.

Abbreviation: API, Asian/Pacific Islander (aggregated).

^a^ Early stage includes in situ and localized tumors.

### 3.1 Incidence rates and IRRs

There were marked differences in stage‐specific CRC IRs (Table 2, Figure S1) and IRRs (Table S1) across racial/ethnic groups. Over the most recent time period (2015–2019), Black and AIAN patients had higher CRC IRs compared to Whites across all disease stages, and these differences were most pronounced for distant stage CRC. For example, IRs of early (IRR = 1.18; 95% CI = 1.14–1.22), regional (IRR = 1.19; 95% CI = 1.13–1.21), and distant (IRR = 1.57; 95% CI = 1.50–1.63) stage CRC among Black patients remained 18%, 19%, and 54% higher (respectively) compared to White patients in the 2015–2019 period and significantly so. In contrast, Hispanic patients had significantly lower IRs for early stage and distant stage CRC compared to White counterparts, across all time periods. API patients, when aggregated, had lower stage‐specific IRs compared to White counterparts, but when disaggregated, Pacific Islander patients had 20% higher IRs of early stage (IRR = 1.20; 95% CI = 1.03–1.40), 27% higher IRs of regional stage (IRR = 1.27; 95% CI = 1.07–1.50), and 67% higher IRs of distant stage (IRR = 1.67, 95% CI = 1.36–2.20) from 2010 to 2014. For comparison, East Asian, Southeast Asian, and South Asian subgroups had similar or lower CRC IRs compared to White counterparts across all time points. Similar IR and IRRS were observed when stratified by subsite (Tables S2–S5).

**TABLE 2:** Trends in stage‐specific incidence and proportional stage distribution of colorectal cancer by race/ethnicity from 2000 to 2019.

	Age‐adjusted IR (per 100,000)						Proportional stage distribution					
Race/stage^a^	2000–2004	2005–2009	2010–2014	2015–2019	APC	*p* value	2000–2004	2005–2009	2010–2014	2015–2019	AD^c^	*p* value^b^ (trend)
White												
Early	56.7	48.5	38.0	31.4	−3.9	<0.001	46.2%	46.6%	44.0%	40.3%	−5.9	<0.001
Regional	43.0	35.2	30.0	28.0	−2.9	<0.001	35.1%	34.0%	34.9%	36.0%	+0.9	<0.001
Distant	19.9	18.0	16.4	16.1	−1.4	<0.001	16.3%	17.5%	19.2%	20.7%	+4.4	<0.001
Black												
Early	61.7	62.3	49.2	37.1	−3.5	<0.001	40.4%	43.6%	41.6%	37.6%	−2.8	<0.001
Regional	53.0	46.4	37.5	32.9	−3.2	<0.001	34.7%	32.9%	32.0%	33.5%	−1.2	0.038
Distant	32.6	29.6	27.3	25.2	−1.7	<0.001	21.6%	21.2%	23.8%	25.8%	+4.2	<0.001
Hispanic												
Early	39.6	37.7	33.1	27.9	−2.4	<0.001	42.3%	42.5%	41.8%	37.6%	−4.8	<0.001
Regional	34.6	31.6	27.5	27.8	−1.5	<0.001	37.1%	36.0%	35.2%	37.6%	+0.6	0.272
Distant	16.6	16.3	15.3	15.1	−0.7	0.001	18.2%	18.8%	19.9%	20.6%	+2.4	<0.001
American Indian												
Early	41.0	42.4	42.4	36.1	−0.9	0.145	42.0%	44.5%	40.8%	38.1%	−3.9	0.046
Regional	37.3	32.3	37.0	34.0	−0.5	0.463	38.2%	33.3%	35.7%	36.2%	−2.0	0.825
Distant	17.4	19.0	21.7	21.1	+0.7	0.292	17.9%	21.1%	21.3%	22.5%	+4.6	0.058
API												
Early	42.2	39.9	34.7	27.0	−2.9	<0.001	42.6%	43.8%	43.1%	38.4%	−4.2	<0.001
Regional	38.4	32.9	28.6	27.2	−2.3	<0.001	38.9%	36.5%	35.8%	38.7%	−0.1	0.955
Distant	16.4	15.5	14.2	13.3	−1.3	<0.001	16.7%	17.5%	18.0%	19.0%	+2.3	<0.001
East Asian												
Early	45.7	41.2	34.6	–^d^	−2.8	<0.001	42.6%	43.7%	42.8%	38.1%	−4.5	<0.001
Regional	42.1	35.9	28.5	–^d^	−3.7	<0.001	39.4%	37.8%	36.6%	39.8%	+0.4	0.950
Distant	17.4	15.4	13.3	–^d^	−2.3	0.002	16.2%	16.4%	17.4%	18.5%	2.4	0.006
Southeast Asian												
Early	34.0	36.2	33.4	–^d^	−0.1	0.846	40.9%	42.9%	41.9%	35.7%	−5.2	<0.001
Regional	33.1	29.6	27.7	–^d^	−1.9	0.003	39.2%	36.7%	36.2%	38.7%	−0.5	0.901
Distant	14.8	15.2	14.5	–^d^	−0.5	0.340	18.1%	18.5%	18.9%	21.5%	+3.4	0.002
South Asian												
Early	19.6	17.3	17.6	–^d^	−1.1	0.103	45.1%	42.1%	41.8%	39.8%	−5.3	0.692
Regional	15.9	15.3	15.7	–^d^	−1.3	0.351	36.6%	37.8%	37.4%	41.0%	+4.4	0.481
Distant	6.5	6.7	7.6	–^d^	+2.5	0.137	15.0%	16.5%	18.0%	15.5%	−0.5	0.775
Pacific Islander												
Early	54.7	46.6	42.1	–^d^	−1.9	0.126	45.9%	40.5%	38.9%	36.6%	−9.3	0.004
Regional	44.4	31.8	35.6	–^d^	−2.1	0.121	36.5%	31.2%	34.2%	36.9%	+0.4	0.463
Distant	19.5	30.2	25.4	–^d^	+1.1	0.594	16.3%	26.0%	24.0%	23.7%	+7.4	0.049

Abbreviations: AD, absolute difference; APC, annual percent change; API, Asian or Pacific Islander (aggregate); IR, incidence rate.

^a^ Early stage includes in‐situ and localized tumors.

^b^ *p* value (trend) for chi‐squared test for linear trend in proportions across time periods.

^c^ Absolute difference between 2000–2004 proportion value and 2015–2019 proportion value for each respective disease stage.

^d^ IRs and IR trends for API subgroups not available for the most recent time period (2015–2019).

### 3.2 Trends in CRC IRS

With regard to trends over time, IRs of early, regional, and distant CRC declined across most racial/ethnic groups (Table 2). Declines in CRC IRs were most pronounced for early stage and regional stage CRC with slower and smaller declines in distant stage CRC. For example, IRs of early and regional stage CRC significantly decreased among White, Black, Hispanic, East Asian, and Southeast Asian patients by approximately −1.5% to −3.9% per year. Smaller declines in distant stage IRs were observed, with significant decreases for White, Black, Hispanic, and East Asian patients by approximately −0.7% to −2.3% per year. Stage‐specific trends in CRC IRS for AIAN, South Asian, and Pacific Islander patients were not statistically significant, likely due to small sample sizes. When examined by stage and subsite, declines in IRs most pronounced for early and regional stage tumors at each subsite for most groups (Tables S2–S5).

### 3.3 Changes in CRC stage distribution

In the most recent time period from 2015 to 2019, the proportion of distant stage CRC was highest among Black (25.8%) and Pacific Islander (23.7%) patients and lowest among South Asian (15.5%) patients (Table 2). When stratified by subsite, Black patients had the highest proportion of distant disease across all subsites in the 2015–2019 period (proximal, 23.9%; distal, 24.3%, and rectum, 26%).

Analysis of shifts in distribution of disease stage at diagnosis over time revealed substantial declines in the proportion of CRC cases detected at early stages concomitant with substantial increases in proportion distant stage CRC across nearly all racial/ethnic groups from 2000–2004 to 2015–2019 (Table 2). There was a small, yet statistically significant increase in regional stage CRC in White patients (from 35.1% to 36.0%, *p* < 0.001) from 2000–2004 to 2015–2019, whereas the proportion CRCs diagnosed at regional stage remained statistically stable among Black, Hispanic, AIAN, and aggregate API groups.

However, when API patients were examined by subgroup, heterogeneity in stage distribution shifts was observed. For example, East Asian, Southeast Asian, and Pacific Islander patients showed a significant decline in proportion CRCs detected at early stages from 2000–2004 to 2015–2019 periods, concomitant with a significant increase in proportion of distant stage CRCs, and no significant changes in distribution of regional disease. In contrast, no significant differences in stage distribution by time period were observed among South Asian and Other Asian subgroups. When examined by subsite, troubling shifts in stage distribution (i.e., decreased proportion of early stage with increased proportion of distant stage tumors) were most pronounced within the distal and rectal subsites (Tables S2–S5).

### 3.4 Factors associated with distant stage CRC

In multivariable logistic regression analysis, Black (OR = 1.22, 95% CI: 1.19–1.25), American Indian (OR = 1.16, 95% CI: 1.06–1.27), and Hispanic (OR = 1.03, 95% CI: 1.001–1.06) patients were more likely to be diagnosed with CRC at distant stages compared with White patients (Table 3). No significant differences in likelihood of distant stage CRC were observed between White and API patients when studied in aggregate (OR = 0.98, 95% CI: 0.95–1.01).

**TABLE 3:** Predictors of distant stage colorectal cancer by race ethnicity, SEER 17 2000–2019.

Characteristic	Univariate		Multivariable	
Race/ethnicity	OR (95% CI)	*p* value	OR (95% CI)	*p* value
White	1.00 (Ref.)		1.00 (Ref.)	
Black	1.34 (1.31–1.37)	<0.001	1.22 (1.19–1.25)	<0.001
Hispanic	1.08 (1.06–1.11)	<0.001	1.03 (1.01–1.06)	0.030
American Indian	1.18 (1.08–1.30)	<0.001	1.16 (1.06–1.27)	0.002
API	0.97 (0.94–1.01)	0.051	0.98 (0.95–1.01)	0.287
East Asian	0.92 (0.88–0.96)	<0.001	0.95 (0.91–0.99)	0.042
Southeast Asian	1.07 (1.02–1.13)	0.006	1.07 (1.02–1.13)	0.006
South Asian	0.88 (0.77–1.00)	0.042	0.89 (0.78–1.01)	0.078
Pacific Islander	1.33 (1.20–1.47)	<0.001	1.28 (1.16–1.42)	<0.001
Sex				
Female	1.00 (Ref.)		1.00 (Ref.)	
Male	1.02 (1.00–1.03)	0.049	1.05 (1.03–1.06)	<0.001
Age at diagnosis (years)				
50–54	1.00 (Ref.)		1.00 (Ref.)	
55–59	1.10 (1.07–1.13)	<0.001	1.09 (1.06–1.12)	<0.001
60–64	1.05 (1.02–1.08)	<0.001	1.05 (1.02–1.08)	0.001
65–69	0.89 (0.87–0.92)	<0.001	0.89 (0.87–0.92)	<0.001
70–74	0.84 (0.82–0.86)	<0.001	0.84 (0.82–0.87)	<0.001
Marital status				
Married	1.00 (Ref.)		1.00 (Ref.)	
Divorced/separated	1.32 (1.28–1.35)	<0.001	1.27 (1.24–1.30)	<0.001
Single	1.45 (1.42–1.48)	<0.001	1.34 (1.31–1.37)	<0.001
Unmarried/domestic partner	1.44 (1.21–1.72)	<0.001	1.27 (1.07–1.52)	0.007
Widowed	1.15 (1.11–1.18)	<0.001	1.21 (1.18–1.25)	<0.001
Unknown	0.74 (0.71–0.77)	<0.001	0.72 (0.69–0.75)	<0.001
Median income				
≥$75,000	1.00 (Ref.)		1.00 (Ref.)	
$65,000–$74,999	1.03 (1.01–1.06)	0.004	1.01 (0.99–1.04)	0.254
$55,0000–$64,999	1.02 (1.00–1.05)	0.035	1.00 (0.98–1.02)	0.926
<$55,000	1.09 (1.06–1.11)	<0.001	1.03 (1.01–1.05)	0.017
Missing	0.60 (0.26–1.41)	0.245	0.87 (0.36–2.13)	0.659
Subsite				
Distal	1.00 (Ref.)		1.00 (Ref.)	
Proximal	1.02 (1.01–1.05)	0.022	1.02 (1.01–1.04)	0.040
Rectum	1.01 (0.98–1.02)	0.999	0.99 (0.96–1.01)	0.178
Colon, NOS	4.17 (3.99–4.36)	<0.001	4.18 (4.00–4.37)	<0.001
Diagnosis year				
2000–2004	1.00 (Ref.)		1.00 (Ref.)	
2005–2009	1.07 (1.05–1.10)	<0.001	1.05 (1.03–1.08)	<0.001
2010–2014	1.19 (1.16–1.22)	<0.001	1.16 (1.13–1.19)	<0.001
2015–2019	1.29 (1.27–1.33)	<0.001	1.25 (1.22–1.28)	<0.001

Abbreviations: API, Asian/Pacific Islander (aggregate); CI, confidence interval; OR, odds ratio.

When examined by API subgroup, Pacific Islander (OR = 1.28, 95% CI: 1.16–1.23) and Southeast Asian (OR = 1.07, 95% CI: 1.02–1.13) patients were more likely to be diagnosed with distant stage CRC compared with White patients, as observed for Black, AIAN, and Hispanic patients. In contrast, East Asian patients were significantly less likely to be diagnosed with distant stage CRC (OR = 0.95, 95% CI: 0.91–0.99) than their White counterparts, and no significant differences in likelihood of distant stage CRC were observed between South Asian and White patients.

Additionally, likelihood of distant stage diagnosis increased over time, as those diagnosed with CRC in the most recent time period (2015–2019) were 25% more likely to be diagnosed at distant stages compared to those diagnosed at the beginning of the study period in years 2000–2004 (OR = 1.25, 95% CI: 1.23–1.28). Other factors such as younger age, male sex, lower median, not being married, and tumors originating in the proximal colon were also associated with a higher likelihood of distant stage presentation.

### 3.5 Survival analysis

**FIGURE 1:**
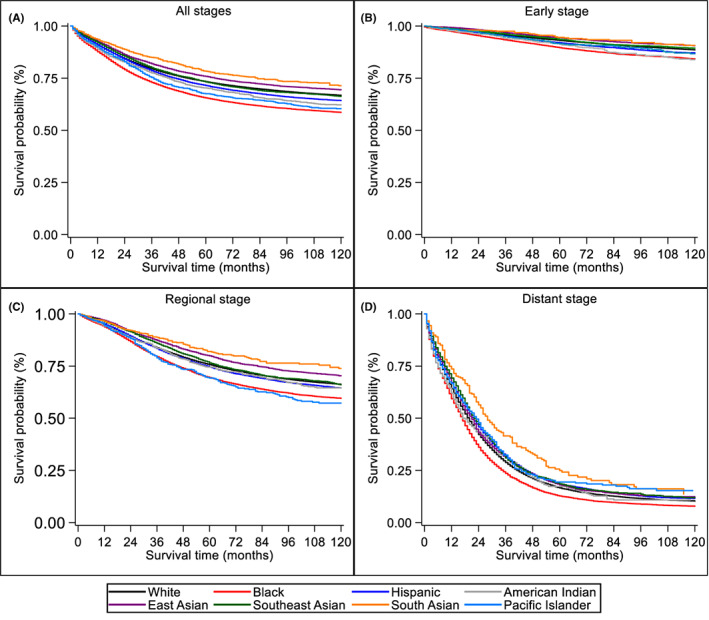
Unadjusted Kaplan–Meier estimates of cause‐specific survival among disaggregated race/ethnic groups for (A) all stages combined (B) early stage (C) regional stage, and (D) distant stage.

In multivariable Cox regression analysis (Table 4, Figure 1A), Black (HR = 1.17, 95% CI: 1.14–1.19), AIAN (HR = 1.10, 95% CI:1.03–1.18), and Pacific Islander (HR = 1.17, 95% CI: 1.08–1.27) patients experienced worse CSS compared to White counterparts, whereas East Asian and South Asian patients experienced better CSS (HR = 0.96, 95% CI: 0.93–0.99 and HR = 0.81, 95% CI: 0.72–0.90, respectively). Conversely, there were no significant differences in CSS among Hispanic and Southeast Asian patients. Other factors such as male sex, older age at diagnosis, earlier year of diagnosis, lower median income, not being married, tumor location in the proximal colon or rectum subsites were also predictive of worse CSS.

**TABLE 4:** Multivariable adjusted^a^ hazard ratio (HR) and 95% confidence interval (CI) estimates for CSS in patients with CRC adenocarcinoma, 2000–2019.

Characteristic	Deaths	HR (95% CI)	*p* value
Race/ethnicity			
White	68,226	1.00 (Ref.)	
Black	14,833	1.17 (1.14–1.19)	<0.001
Hispanic	10,934	0.99 (0.98–1.02)	0.934
American Indian	795	1.10 (1.03–1.18)	0.007
API	7473	0.97 (0.94–0.99)	<0.001
East Asian	3384	0.96 (0.93–0.99)	0.018
Southeast Asian	2633	1.01 (0.97–1.05)	0.730
South Asian	345	0.81 (0.72–0.90)	<0.001
Pacific Islander	623	1.17 (1.08–1.27)	<0.001
Sex			
Female	42,483	1.00 (Ref.)	
Male	59,853	1.12 (1.11–1.14)	<0.001
Age at diagnosis			
50–54	14,585	1.00 (Ref.)	
55–59	18,323	1.08 (1.06–1.11)	<0.001
60–64	21,282	1.17 (1.14–1.19)	<0.001
65–69	23,340	1.24 (1.22–1.27)	<0.001
70–74	24,806	1.45 (1.42–1.48)	<0.001
Marital status			
Married	56,242	1.00 (Ref.)	
Divorced/separated	13,692	1.22 (1.19–1.24)	<0.001
Single	17,531	1.28 (1.26–1.30)	<0.001
Unmarried/domestic partner	146	1.22 (1.04–1.44)	0.015
Widowed	9693	1.23 (1.20–1.26)	<0.001
Unknown	5032	1.03 (1.00–1.06)	0.031
Median income			
≥$75,000	28,287	1.00 (Ref.)	
$65,000–$74,999	22,459	1.09 (1.07–1.11)	<0.001
$55,000–$64,999	25,469	1.12 (1.11–1.15)	<0.001
<$55,000	26,110	1.21 (1.18–1.23)	<0.001
Unknown	11	0.88 (0.47–1.63)	0.684
Subsite			
Distal colon	25,646	1.00 (Ref.)	
Proximal colon	38,643	1.20 (1.18–1.22)	<0.001
Rectum	33,847	1.09 (1.07–1.11)	<0.001
Colon, NOS	4200	1.35 (1.31–1.67)	<0.001
Surgery			
Yes	75,276	1.00 (Ref.)	
No/unknown	27,060	2.43 (2.39–2.47)	<0.001
Diagnosis year			
2000–2004	31,872	1.00 (Ref.)	
2005–2009	28,919	0.85 (0.84–0.86)	<0.001
2010–2014	26,649	0.77 (0.76–0.79)	<0.001
2015–2019	14,896	0.67 (0.66–0.68)	<0.001

*Note*: Patients listed as “other Asian” were included in the aggregate API group (deaths = 488).

Abbreviations: API, Asian/Pacific Islander (aggregate of all subgroups); CI, confidence interval; HR, hazards ratio.

^a^ Stratified by stage at diagnosis; adjusted for all variables in the table.

In separate models for stage at diagnosis (Table 5, Figure 1B–D), Black patients had significantly worse CSS than Whites across all disease stages, and the survival disparity was most pronounced for early stage (HR = 1.38, 95% CI: 1.32–1.44) and regional stage (HR = 1.22, 95% CI: 1.18–1.26) CRC. Hispanic and Pacific Islander patients with early stage or regional stage (but not distant stage) disease also experienced a 5%–45% higher risk of CRC mortality than White counterparts diagnosed at similar stages. When diagnosed at distant stages, however, Hispanic patients had better CSS compared to White patients with distant CRC (HR = 0.93, 95% CI: 0.90–0.95). Although AIAN patients with early stage CRC experienced worse survival (HR = 1.47, 95% CI: 1.24–1.75), AIAN patients with regional or distant stage disease showed no significant differences in CSS compared to White patients. At every disease stage, East Asian, Southeast Asian, and South Asian subgroups had equivalent or better CSS than White patients.

**TABLE 5:** Multivariable adjusted^a^ hazard ratio (HR) and 95% confidence interval (CI) estimates for CSS in patients with CRC adenocarcinoma by stage at diagnosis, 2000–2019.

	Early stage^b^			Regional stage			Distant stage		
Race/ethnicity	Deaths	HR (95% CI)	*p* value	Deaths	HR (95% CI)	*p* value	Deaths	HR (95% CI)	*p* value
White	10,481	1.00 (Ref.)		23,228	1.00 (Ref.)		32,515	1.00 (Ref.)	
Black	2235	1.38 (1.31–1.44)	<0.001	4613	1.22 (1.18–1.26)	<0.001	7447	1.07 (1.04–1.10)	<0.001
Hispanic	1554	1.15 (1.09–1.21)	<0.001	3721	1.05 (1.02–1.09)	0.002	5250	0.93 (0.90–0.95)	<0.001
American Indian	129	1.47 (1.24–1.75)	<0.001	248	1.07 (0.94–1.21)	0.294	397	1.05 (0.95–1.16)	0.316
API	944	0.94 (0.88–1.00)	0.068	2610	0.95 (0.91–0.99)	0.022	3626	0.97 (0.94–1.01	0.116
East Asian	428	0.89 (0.81–0.98)	0.018	1204	0.90 (0.85–0.96)	<0.001	1625	1.02 (0.97–1.07)	0.473
Southeast Asian	328	1.05 (0.94–1.17)	0.386	934	1.06 (1.00–1.14)	0.063	1265	0.95 (0.90–1.00)	0.071
South Asian	44	0.88 (0.66–1.19)	0.404	115	0.81 (0.67–0.97)	0.025	173	0.78 (0.67–0.91)	0.001
Pacific Islander	81	1.39 (1.12–1.74)	0.003	220	1.45 (1.27–1.66)	<0.001	301	0.96 (0.86–1.08)	0.528
Sex									
Female	5821	1.00 (Ref.)		14,458	1.00 (Ref.)		20,966	1.00 (Ref.)	
Male	9530	1.28 (1.24–1.33)	<0.001	19,981	1.16 (1.13–1.19)	<0.001	28,306	1.05 (1.04–1.07)	<0.001
Age at diagnosis									
50–54	1666	1.00 (Ref.)		4683	1.00 (Ref.)		7845	1.00 (Ref.)	
55–59	2253	1.22 (1.15–1.30)	<0.001	5779	1.09 (1.04–1.13)	<0.001	9745	1.04 (1.01–1.08)	0.005
60–64	2850	1.37 (1.29–1.46)	<0.001	6923	1.19 (1.14–1.23)	<0.001	10,820	1.10 (1.07–1.14)	<0.001
65–69	3854	1.63 (1.54–1.73)	<0.001	8083	1.27 (1.22–1.31)	<0.001	10,662	1.15 (1.11–1.18)	<0.001
70–74	4728	2.11 (1.99–2.23)	<0.001	8971	1.47 (1.42–1.53)	<0.001	10,200	1.29 (1.25–1.33)	<0.001
Marital status									
Married	8624	1.00 (Ref.)		19,349	1.00 (Ref.)		26,912	1.00 (Ref.)	
Divorced/separated	1885	1.36 (1.30–1.43)	<0.001	4553	1.28 (1.24–1.33)	<0.001	6862	1.13 (1.10–1.16)	<0.001
Single	2282	1.42 (1.35–1.48)	<0.001	5664	1.37 (1329–1.41)	<0.001	8988	1.18 (1.16–1.21)	<0.001
Unmarried/domestic partner	14	1.29 (0.76–2.18)	0.344	44	1.42 (1.05–1.90)	0.021	87	1.14 (0.92–1.41)	0.223
Widowed	1554	1.36 (1.28–1.44)	<0.001	3468	1.29 (1.24–1.34)	<0.001	4366	1.16 (1.13–1.20)	<0.001
Unknown	992	0.92 (0.86–0.99)	0.016	1361	1.15 (1.09–1.21)	<0.001	2057	1.04 (0.99–1.08)	0.101
Median income									
≥$75,000	3875	1.00 (Ref.)		9489	1.00 (Ref.)		14,061	1.00 (Ref.)	
$65,000–$74,999	3241	1.13 (1.08–1.19)	<0.001	7596	1.10 (1.06–1.13)	<0.001	10,899	1.08 (1.05–1.11)	<0.001
$55,000–$64,999	3935	1.22 (1.17–1.28)	<0.001	8760	1.15 (1.11–1.18)	<0.001	11,932	1.10 (1.07–1.12)	<0.001
<$55,000	4298	1.46 (1.39–1.52)	<0.001	8593	1.22 (1.18–1.25)	<0.001	12,377	1.14 (1.11–1.17)	<0.001
Unknown	2	0.65 (0.16–2.59)	0.586	1	0.70 (0.10–4.95)	0.720	3	0.94 (0.30–2.92)	0.921
Subsite									
Distal colon	3984	1.00 (Ref.)		7980	1.00 (Ref.)		13,067	1.00 (Ref.)	
Proximal colon	4907	1.05 (1.01–1.10)	0.021	14,045	1.11 (1.08–1.14)	<0.001	18,967	1.32 (1.29–1.35)	<0.001
Rectum	6257	1.57 (1.50–1.63)	<0.001	11,813	1.19 (1.15–1.22)	<0.001	14,271	0.88 (0.86–0.90)	<0.001
Colon, NOS	203	1.07 (0.93–1.23)	0.348	601	1.49 (1.37–1.62)	<0.001	2967	1.31 (1.26–1.37)	<0.001
Surgery									
Yes	13,285	1.00 (Ref.)		32,365	1.00 (Ref.)		28,845	1.00 (Ref.)	
No/unknown	2066	4.22 (4.03–4.43)	<0.001	2074	3.24 (3.09–3.39)	<0.001	20,427	2.33 (2.29–2.38)	<0.001
Diagnosis year									
2000–2004	5874	1.00 (Ref.)		12,232	1.00 (Ref.)		12,837	1.00 (Ref.)	
2005–2009	4863	0.90 (0.87–0.94)	<0.001	10,072	0.86 (0.84–0.88)	<0.001	13,119	0.80 (0.78–0.82)	<0.001
2010–2014	3472	0.88 (0.84–0.92)	<0.001	8525	0.79 (0.77–0.81)	<0.001	13,790	0.71 (0.69–0.73)	<0.001
2015–2019	1142	0.74 (0.70–0.79)	<0.001	3610	0.69 (0.66–0.72)	<0.001	9526	0.63 (0.61–0.64)	<0.001

*Note*: Patients listed as “other Asian” were included in the aggregate API group (deaths = 488).

Abbreviations: API, Asian/Pacific Islander (aggregate of all subgroups); CI, confidence interval; HR, hazards ratio.

^a^ Adjusted for all variables in the table.

^b^ Early stage include in‐situ and localized tumors.

## 4 DISCUSSION

Despite reductions in overall CRC IRS over the past two decades, striking disparities by race/ethnicity, stage, and tumor subsite remain. We find that for most racial/ethnic groups, IRs of distant stage CRC have decreased at a slower rate compared to IRs for early stage tumors. As a result, for most groups, distant stage tumors comprise an increasing proportion of all CRC cases, which aligns with findings from previous research.^17^ Moreover, findings suggest that the burden of CRC is not evenly distributed across racial/ethnic groups, with marked disparities in CRC incidence, stage at diagnosis, and CSS by race/ethnicity, especially for certain API subgroups. We found that Black, American Indian, and Hispanic patients were more likely to be diagnosed at distant stages than their White counterparts, whereas API patients, when aggregated as one group, showed no significant differences in likelihood of diagnosis with distant stage CRC. When API patients were disaggregated, however, marked heterogeneity in CRC outcomes between API subgroups emerged. Specifically, Pacific Islander and Southeast Asian subgroups were also more likely to be diagnosed at distant or more advanced stages, as was observed for Black, AIAN, and Hispanic patients, stages of disease that are less amenable to curative treatment and often fatal.

In addition to higher risk of diagnosis with distant stage disease, Black, AIAN, and Pacific Islander patients showed poorer CSS compared to White patients, and these disparities remained after adjusting for key sociodemographic and tumor characteristics. In stage stratified analysis, findings of worse CSS among Black, Hispanic, and Pacific Islander patients persisted even among those diagnosed at earlier disease stages, when treatment is most effective. Differences in CSS by race/ethnicity, especially among patients diagnosed at earlier stages, point to disparities across the cancer care continuum from screening to treatment delivery and follow‐up care.

Although Pacific Islanders are frequently grouped with Asian Americans, we show that disaggregating by subgroup can reveal important differences in CRC incidence, stage, and survival not observable when aggregated into a single group. While several studies have characterized differences in CRC outcomes between Asian American subgroups,^8^, ^18^, ^19^, ^20^, ^21^, ^22^ few report outcomes for Pacific Islanders separately from other Asian subgroups. We find that when API data are disaggregated, significant variations in CRC outcomes across subgroups emerged. Specifically, our findings indicate that Pacific Islanders have distinct risk profiles for CRC that are similar to those of Black and AIAN patients. Additionally, we found that despite having higher risk of diagnosis with distant stage CRC, Southeast Asian patients had similar CSS to White patients. These findings reinforce the importance of disaggregating racial/ethnic data whenever possible to provide a more accurate depiction of progress made toward health equity in cancer.

There are genetic, lifestyle, and environmental factors that may contribute to the observed differences in CRC outcomes across racial and ethnic groups. Although elevated risk of CRC can be inherited, the majority (70%–75%) of CRC cases are sporadic and occur in people without genetic predisposition or family history of the disease.^23^, ^24^ Lifestyle and environmental factors including alcohol consumption and tobacco use, high intake of red and processed meat, obesity, and sedentary lifestyle increase the risk of developing CRC. Although traditional Asian diets are typically rich in antioxidant and anti‐inflammatory compounds proposed to be protective against CRC, acculturation and adoption of “Western” diets (high intake of red meat and saturated fats and low intake of fiber) and lifestyle behaviors (low physical activity, sedentary habits) may explain some of the variability in CRC outcomes between different API subgroups.^25^, ^26^ Differences in neighborhood and built environments can also contribute to health disparities. For example, Black and Hispanic populations are more likely to live in neighborhoods that lack access to healthy food options, recreational spaces for physical activity, and healthcare facilities to receive preventive services, all of which may contribute to the poorer CRC outcomes observed.^27^, ^28^

While lifestyle and environmental factors impact risk of developing the disease, the observed differences in disease stage and survival by race/ethnicity likely result from inequities in access to and knowledge of CRC screening and treatment services. Racial/ethnic minority populations have consistently lower CRC screening rates than White counterparts, which, in turn, contribute to later stage diagnosis and subsequent poorer survival in minority patients.^2^, ^29^, ^30^, ^31^ Racial/ethnic minority populations also experience higher rates of poverty, are more likely to be uninsured, and are less likely to have a usual source of care, all of which have been linked to lower utilization of CRC screening and delayed diagnosis.^32^, ^33^, ^34^ Lack of knowledge about CRC and CRC screening modalities, lack of physician's recommendation for screening, fear of the screening process, fear of results, cancer fatalism, and distrust of the medical system have also been identified as powerful predictors of low CRC screening adherence in API and other minority populations.^35^, ^36^, ^37^ English language proficiency and knowledge of US healthcare systems are additional barriers to CRC screening in Asian and Hispanic immigrant groups.^38^ Lack of culturally appropriate cancer education resources, unintended bias exercised by providers, discrimination and structural racism experienced by patients are also likely key contributors to the observed differences in CRC burden among minority groups.

Although the exact mechanisms contributing to the observed racial/ethnic disparities in CRC are not well understood, there is growing recognition that health disparities emerge and persist through multiple domains of influence that include individual (e.g., health behaviors, family history, and genetic risk), community and environmental (e.g., residential segregation, access to healthy food choices, access to screening and quality healthcare), and system‐level factors (e.g., laws and policies that impact access to, receipt, and quality of cancer care).^39^, ^40^ Use of patient navigation programs has been shown to substantially improve CRC screening utilization for minority populations^41^; however, tailored interventions that target the many multilevel causes of CRC disparities are needed.

In 2021, The United States Preventive Services Task Force expanded the recommended ages for CRC screening to 45 to 75 years (previously 50–75 years).^42^ An estimated 20 million Americans between 45 and 49 years of age are now newly eligible for routine CRC screening, through insurance coverage for preventive services, as mandated by the Affordable Care Act.^43^, ^44^ Despite this, CRC screening continues to be underutilized by underserved groups most likely to benefit from early detection, and there is concern that expanding the screening‐eligible population may divert important resources away from populations at greatest risk of CRC who are most likely to benefit from screening and early detection. Future studies should monitor the effect of revised screening guidelines on disparities in CRC incidence, stage at diagnosis, and survival to ensure equal benefit across all racial/ethnic subpopulations.

Our study has several limitations.^45^ First, patients listed as “other Asian” were excluded from subgroup analysis due to unavailability of incidence data in the SEER 9 database. Also, SEER does not include information on disease risk factors such as diet, obesity, smoking or alcohol consumption, family history, comorbidities, social support, acculturation, and other factors related to healthcare access that could inform our study findings. SEER also does not provide any data pertaining to the modality or frequency of CRC screening, as well as information on genetic testing. Furthermore, we were unable to differentiate Hispanic subgroups, such as Mexican, Cuban, Puerto Rican, and other Central and South American countries. We were also unable to disaggregate AIAN groups by tribal affiliation, as this information is not available in SEER.

## 5 CONCLUSION

Our study provides an updated and broader examination of CRC disparities by race/ethnicity and demonstrates the extent to which aggregating heterogenous populations masks significant variability in both the CRC outcomes within subgroups and its burden. Improved understanding of the CRC disparities among API groups requires collection of data for these groups, and probably for other aggregated ethnic groups (i.e., Hispanics) to allow critical comparisons with majority racial/ethnic populations but also within heterogenous subgroups.

## AUTHOR CONTRIBUTIONS

**Kristin M. Primm:** Conceptualization (lead); formal analysis (lead); investigation (lead); methodology (lead); visualization (lead); writing – original draft (lead); writing – review and editing (lead). **Andrea Joyce Malabay:** Formal analysis (supporting); investigation (supporting); visualization (supporting); writing – original draft (supporting); writing – review and editing (equal). **Taylor Curry:** Formal analysis (supporting); investigation (supporting); visualization (supporting); writing – original draft (supporting); writing – review and editing (equal). **Shine Chang:** Conceptualization (equal); formal analysis (supporting); funding acquisition (lead); investigation (equal); methodology (supporting); resources (equal); supervision (lead); writing – original draft (equal); writing – review and editing (equal).

## FUNDING INFORMATION

This work was supported by the Cancer Prevention and Research Institute of Texas (RP170259 and R25CA056452).

## CONFLICT OF INTEREST STATEMENT

The authors declare no potential conflicts of interest.

## Supporting information


Data S1.

## Data Availability

The data presented in this study are available publicly on the Surveillance, Epidemiology, and End Results Program website (https://seer.cancer.gov).

## References

[1] Siegel RL , Miller KD , Goding Sauer A , et al. Colorectal cancer statistics, 2020. CA Cancer J Clin. 2020;70(3):145‐164. 32133645 10.3322/caac.21601

[2] Lansdorp‐Vogelaar I , Kuntz KM , Knudsen AB , van Ballegooijen M , Zauber AG , Jemal A . Contribution of screening and survival differences to racial disparities in colorectal cancer rates. Cancer Epidemiol Biomarkers Prev. 2012;21(5):728‐736. 22514249 10.1158/1055-9965.EPI-12-0023 PMC3531983

[3] May FP , Glenn BA , Crespi CM , Ponce N , Spiegel BMR , Bastani R . Decreasing black‐white disparities in colorectal cancer incidence and stage at presentation in the United States. Cancer Epidemiol Biomarkers Prev. 2017;26(5):762‐768. 28035021 10.1158/1055-9965.EPI-16-0834 PMC5413405

[4] Oh DL , Santiago‐Rodríguez EJ , Canchola AJ , Ellis L , Tao L , Gomez SL . Changes in colorectal cancer 5‐year survival disparities in California, 1997–2014. Cancer Epidemiol Biomarkers Prev. 2020;29(6):1154‐1161. 32371552 10.1158/1055-9965.EPI-19-1544 PMC7269803

[5] Petrick JL , Barber LE , Warren Andersen S , Florio AA , Palmer JR , Rosenberg L . Racial disparities and sex differences in early‐ and late‐onset colorectal cancer incidence, 2001–2018. Front Oncol. 2021;11:734998. 34568072 10.3389/fonc.2021.734998 PMC8459723

[6] Robbins AS , Siegel RL , Jemal A . Racial disparities in stage‐specific colorectal cancer mortality rates from 1985 to 2008. J Clin Oncol. 2012;30(4):401‐405. 22184373 10.1200/JCO.2011.37.5527

[7] Chien C , Morimoto LM , Tom J , Li CI . Differences in colorectal carcinoma stage and survival by race and ethnicity. Cancer. 2005;104(3):629‐639. 15983985 10.1002/cncr.21204

[8] Ellis L , Abrahao R , McKinley M , et al. Colorectal cancer incidence trends by age, stage, and racial/ethnic group in California, 1990–2014. Cancer Epidemiol Biomarkers Prev. 2018;27(9):1011‐1018. 30115679 10.1158/1055-9965.EPI-18-0030

[9] Gu M , Thapa S . Colorectal cancer in the United States and a review of its heterogeneity among Asian American subgroups. Asia Pac J Clin Oncol. 2020;16(4):193‐200. 32129941 10.1111/ajco.13324

[10] Soneji S , Iyer SS , Armstrong K , Asch DA . Racial disparities in stage‐specific colorectal cancer mortality: 1960–2005. Am J Public Health. 2010;100(10):1912‐1916. 20724684 10.2105/AJPH.2009.184192 PMC2936993

[11] Cress RD , Morris C , Ellison GL , Goodman MT . Secular changes in colorectal cancer incidence by subsite, stage at diagnosis, and race/ethnicity, 1992–2001. Cancer. 2006;107(S5):1142‐1152. 16835912 10.1002/cncr.22011

[12] Garcia S , Pruitt SL , Singal AG , Murphy CC . Colorectal cancer incidence among Hispanics and non‐Hispanic whites in the United States. Cancer Causes Control. 2018;29(11):1039‐1046. 30155605 10.1007/s10552-018-1077-1 PMC6628724

[13] Surveillance, Epidemiology, and End Results (SEER) Program . SEER*Stat Database: Incidence–SEER Research Plus Data, 17 Registries, Nov 2021 Sub (2000–2019)–Linked to County Attributes–Total U.S., 1969–2019 Counties, Based on November 2020 Submission, National Cancer Institute, DCCPS, Surveillance Research Program. (https://www.seer.cancer.gov), released April 2021

[14] Carr NJ , McCarthy WF , Sobin LH . Epithelial noncarcinoid tumors and tumor‐like lesions of the appendix. A clinicopathologic study of 184 patients with a multivariate analysis of prognostic factors. Cancer. 1995;75(3):757‐768. 7828125 10.1002/1097-0142(19950201)75:3<757::aid-cncr2820750303>3.0.co;2-f

[15] Surveillance, Epidemiology, and End Results (SEER) Program . SEER*Stat Database: Incidence–SEER 9, Plus Remainder of CA and NJ, Nov 2016 Sub (1990–2014) Detailed API Plus White Non‐Hispanic–Pops Projected from Populations, Based on the November 2016 Submission, National Cancer Institute, DCCPS, Surveillance Research Program. (https://www.seer.cancer.gov), released May 2017

[16] StataCorp . Stata Statistical Software: Release 15. StataCorp LLC; 2017.

[17] Augustus GJ , Roe DJ , Jacobs ET , Lance P , Ellis NA . Is increased colorectal screening effective in preventing distant disease? PLoS ONE. 2018;13(7):e0200462. 30001362 10.1371/journal.pone.0200462 PMC6042755

[18] Gomez SL , Noone A‐M , Lichtensztajn DY , et al. Cancer incidence trends among Asian American populations in the United States, 1990–2008. J Natl Cancer Inst. 2013;105(15):1096‐1110. 23878350 10.1093/jnci/djt157 PMC3735462

[19] Park HM , Woo H , Jung SJ , Jung KW , Shin HR , Shin A . Colorectal cancer incidence in 5 Asian countries by subsite: an analysis of cancer incidence in five continents (1998–2007). Cancer Epidemiol. 2016;45:65‐70. 27716537 10.1016/j.canep.2016.09.012

[20] Kwong SL , Chen MS Jr , Snipes KP , Bal DG , Wright WE . Asian subgroups and cancer incidence and mortality rates in California. Cancer. 2005;104(S12):2975‐2981. 16247792 10.1002/cncr.21511 PMC1810966

[21] McCracken M , Olsen M , Chen MS Jr , et al. Cancer incidence, mortality, and associated risk factors among Asian Americans of Chinese, Filipino, Vietnamese, Korean, and Japanese ethnicities. CA Cancer J Clin. 2007;57(4):190‐205. 17626117 10.3322/canjclin.57.4.190

[22] Miller BA , Chu KC , Hankey BF , Ries LA . Cancer incidence and mortality patterns among specific Asian and Pacific Islander populations in the US. Cancer Causes Control. 2008;19(3):227‐256. 18066673 10.1007/s10552-007-9088-3 PMC2268721

[23] Carethers JM . Racial and ethnic disparities in colorectal cancer incidence and mortality. In: Berger FG, Boland CR , eds. Novel approaches to Colorectal cancer. Advances in Cancer Research. Vol 151. Elsevier; 2021:197‐229. 10.1016/bs.acr.2021.02.007 PMC9069392 34148614

[24] Lichtenstein P , Holm NV , Verkasalo PK , et al. Environmental and heritable factors in the causation of cancer—analyses of cohorts of twins from Sweden, Denmark, and Finland. N Engl J Med. 2000;343(2):78‐85. 10891514 10.1056/NEJM200007133430201

[25] Dermadi D , Valo S , Ollila S , et al. Western diet deregulates bile acid homeostasis, cell proliferation, and tumorigenesis in Colon. Cancer Res. 2017;77(12):3352‐3363. 28416481 10.1158/0008-5472.CAN-16-2860

[26] Mehta RS , Song M , Nishihara R , et al. Dietary patterns and risk of colorectal cancer: analysis by tumor location and molecular subtypes. Gastroenterology. 2017;152(8):1944‐1953. e1941. 28249812 10.1053/j.gastro.2017.02.015 PMC5447483

[27] Fong AJ , Lafaro K , Ituarte PH , Fong Y . Association of living in urban food deserts with mortality from breast and colorectal cancer. Ann Surg Oncol. 2021;28(3):1311‐1319. 32844294 10.1245/s10434-020-09049-6 PMC8046424

[28] Masdor NA , Mohammed Nawi A , Hod R , Wong Z , Makpol S , Chin S‐F . The link between food environment and colorectal cancer: a systematic review. Nutrients. 2022;14(19):3954. 36235610 10.3390/nu14193954 PMC9573320

[29] Andersen SW , Blot WJ , Lipworth L , Steinwandel M , Murff HJ , Zheng W . Association of race and socioeconomic status with colorectal cancer screening, colorectal cancer risk, and mortality in southern US adults. JAMA Netw Open. 2019;2(12):e1917995. 31860105 10.1001/jamanetworkopen.2019.17995 PMC6991213

[30] Burnett‐Hartman AN , Mehta SJ , Zheng Y , et al. Racial/ethnic disparities in colorectal cancer screening across healthcare systems. Am J Prev Med. 2016;51(4):e107‐e115. 27050413 10.1016/j.amepre.2016.02.025 PMC5030113

[31] Laiyemo AO , Doubeni C , Pinsky PF , et al. Race and colorectal cancer disparities: health‐care utilization vs different cancer susceptibilities. J Natl Cancer Inst. 2010;102(8):538‐546. 20357245 10.1093/jnci/djq068 PMC2857802

[32] Beydoun HA , Beydoun MA . Predictors of colorectal cancer screening behaviors among average‐risk older adults in the United States. Cancer Causes Control. 2008;19(4):339‐359. 18085415 10.1007/s10552-007-9100-y

[33] Laiyemo AO , Adebogun AO , Doubeni CA , et al. Influence of provider discussion and specific recommendation on colorectal cancer screening uptake among US adults. Prev Med. 2014;67:1‐5. 24967957 10.1016/j.ypmed.2014.06.022 PMC4167462

[34] Nagelhout E , Comarell K , Samadder N , Wu YP . Barriers to colorectal cancer screening in a racially diverse population served by a safety‐net clinic. J Community Health. 2017;42(4):791‐796. 28168395 10.1007/s10900-017-0319-6 PMC5517041

[35] Morey BN , Valencia C , Lee S . The influence of Asian subgroup and acculturation on colorectal cancer screening knowledge and attitudes among Chinese and Korean Americans. J Cancer Educ. 2022;37(6):1806‐1815. 34106449 10.1007/s13187-021-02042-x PMC8188737

[36] Domingo J‐LB , Chen JJ , Braun KL . Colorectal cancer screening compliance among Asian and Pacific Islander Americans. J Immigr Minor Health. 2018;20(3):584‐593. 28378254 10.1007/s10903-017-0576-6

[37] Maxwell AE , Crespi CM , Antonio CM , Lu P . Explaining disparities in colorectal cancer screening among five Asian ethnic groups: a population‐based study in California. BMC Cancer. 2010;10(1):1‐9. 20482868 10.1186/1471-2407-10-214 PMC2888788

[38] Cataneo JL , Kim TD , Park JJ , Marecik S , Kochar K . Disparities in screening for colorectal cancer based on limited language proficiency. Am Surg. 2022;88(11):2737‐2744. 35642266 10.1177/00031348221105596

[39] Gorin SS , Badr H , Krebs P , Das IP . Multilevel interventions and racial/ethnic health disparities. J Natl Cancer Inst Monogr. 2012;2012(44):100‐111. 22623602 10.1093/jncimonographs/lgs015 PMC3482960

[40] Warnecke RB , Oh A , Breen N , et al. Approaching health disparities from a population perspective: the National Institutes of Health centers for population health and health disparities. Am J Public Health. 2008;98(9):1608‐1615. 18633099 10.2105/AJPH.2006.102525 PMC2509592

[41] Grubbs SS , Polite BN , Carney J Jr , et al. Eliminating racial disparities in colorectal cancer in the real world: it took a village. J Clin Oncol. 2013;31(16):1928‐1930. 23589553 10.1200/JCO.2012.47.8412 PMC3661932

[42] US Preventive Services Task Force , Davidson KW , Barry MJ , Mangione CM , et al. Screening for colorectal cancer: US preventive services task force recommendation statement. JAMA. 2021;325(19):1965‐1977. 34003218 10.1001/jama.2021.6238

[43] Piscitello A , Edwards DK . Estimating the screening‐eligible population size, ages 45–74, at average risk to develop colorectal cancer in the United States. Cancer Prev Res. 2020;13(5):443‐448. 10.1158/1940-6207.CAPR-19-0527 32029430

[44] Rosenbaum S . The patient protection and affordable care act: implications for public health policy and practice. Public Health Rep. 2011;126(1):130‐135. 21337939 10.1177/003335491112600118 PMC3001814

[45] Yu JB , Gross CP , Wilson LD , Smith BD . NCI SEER public‐use data: applications and limitations in oncology research. Oncology (Williston). 2009;23(3):288‐295. 19418830

