# Supplementary material for: Who, where, when: Colorectal cancer disparities by race and ethnicity, subsite, and stage
Source: Cancer Med. 2023 May 22;12(13):14767–80. doi: 10.1002/cam4.6105 (PMC10358189; doi:10.1002/cam4.6105)
Supplement: Supplementary file 1 — Data S1. [file CAM4-12-14767-s001.docx]

SUPPLEMENTAL METHODS

This study utilized two SEER databases to derive IR estimates for this analysis. Annual CRC IRs for White, Black, Hispanic, AIAN, and aggregate API patients were calculated from the SEER 17 incidence dataset (November 2021 submission). Because incidence data for specific API subgroups are not available in the standard SEER 17 incidence dataset, the specialized SEER 9 incidence database for detailed Asian/Pacific Islander groups was used to calculate age adjusted IRs for API subgroups. SEER`s specialized API database has not been updated for the most recent SEER data release (November 2021 submission), therefore trends in IRs for API subgroups are presented for years 2000-2014 (calculated from the specialized SEER 9 database), reflecting the most recent data available at the time of analysis.

Baseline characteristics for each patient were extracted from the SEER 17 database using the “Case Listing Session” of SEER*Stat software. Variables included age at diagnosis, year of diagnosis, race/ethnicity, sex, marital status, county level median household income, tumor site, stage at diagnosis, vital status, cause of death, survival time, and treatment via surgery.

To determine whether proportions of cancer stage are shifting over time, cancer stage proportions (i.e., percent contribution of each cancer stage to overall incidence) are presented by time period (5-year groupings) and by race/ethnicity. Chi square tests for trend were used to assess trends in stage distribution in the consecutive time periods. Because cases with unknown stage impact staging shifts, tumors with unknown stage were included in stage distribution trend analysis.

Univariate and multivariable Logistic regression models were used to assess the association of race/ethnicity and distant stage diagnosis. The dependent variable for the logistic regression analyses was distant stage diagnosis (versus early, regional, and unknown stage). Multivariable logistic regression models included adjustments for age at diagnosis (50-54, 55-59, 60-64, 65-69, or 70-74 years), sex, marital status (married, not married, unknown/missing), county-level median household income ($70,000, $50,000-$74,999, <$50,000, unknown/missing), tumor subsite (proximal, distal, rectum, and colon NOS) and diagnosis year.

Multivariable cox proportional hazards models included all covariates described above and additionally adjusted for treatment via surgery (yes, no/unknown), and diagnosis stage. The proportional hazards assumption was evaluated using Schoenfeld residuals and log–log survival function plots. There was evidence of a violation of the proportional hazards assumption for stage at diagnosis; therefore, stage stratified Cox models are presented.

SUPPLEMENTAL TABLES AND FIGURES

Supplemental Table S1. Five-year age-adjusted IRRs of colorectal cancer by stage at diagnosis, race and ethnicity, and time period

|  |  | 2000-2004  IRR (95% CI) | 2005-2009  IRR (95% CI) | 2010-2014  IRR (95% CI) | 2015-2019  IRR (95% CI) |
| --- | --- | --- | --- | --- | --- |
| Early^a^ | Black | 1.09 (1.05, 1.13) | 1.28 (1.25, 1.32) | 1.29 (1.25, 1.33) | 1.18 (1.14, 1.22) |
|  | Hispanic | 0.70 (0.67, 0.72) | 0.78 (0.75, 0.80) | 0.87 (0.84, 0.90) | 0.89 (0.86, 0.92) |
|  | American Indian | 0.72 (0.63, 0.83) | 0.87 (0.77, 0.99) | 1.12 (0.99, 1.25) | 1.15 (1.03, 1.28) |
|  | API | 0.75 (0.72, 0.78) | 0.82 (0.79, 0.85) | 0.91 (0.88, 0.94) | 0.86 (0.83, 0.89) |
|  | East Asian | 0.84 (0.80, 0.89) | 0.90 (0.85, 0.94) | 0.99 (0.93, 1.04) | ~ |
|  | South Asian | 0.36 (0.29, 0.44) | 0.38 (0.32, 0.45) | 0.50 (0.43, 0.58) | ~ |
|  | Southeast Asian | 0.63 (0.58, 0.67) | 0.79 (0.74, 0.84) | 0.95 (0.90, 1.01) | ~ |
|  | Pacific Islander | 1.01 (0.85, 1.18) | 1.01 (0.86, 1.18) | 1.20 (1.03, 1.40) | ~ |
| Regional | Black | 1.23 (1.19, 1.28) | 1.32 (1.27, 1.36) | 1.25 (1.21, 1.30) | 1.19 (1.13, 1.21) |
|  | Hispanic | 0.81 (0.77, 0.84) | 0.90 (0.86, 0.93) | 0.92 (0.88, 0.95) | 0.99 (0.96, 1.03) |
|  | American Indian | 0.87 (0.75, 1.00) | 0.92 (0.79, 1.06) | 1.23 (1.09, 1.39) | 1.22 (1.08, 1.36) |
|  | API | 0.89 (0.86, 0.93) | 0.94 (0.90, 0.97) | 0.95 (0.92, 0.99) | 0.97 (0.94, 1.01) |
|  | East Asian | 1.01 (0.96, 1.07) | 1.07 (1.01, 1.13) | 1.02 (0.96, 1.08) | ~ |
|  | South Asian | 0.38 (0.31, 0.47) | 0.45 (0.38, 0.54) | 0.56 (0.48, 0.66) | ~ |
|  | Southeast Asian | 0.80 (0.74, 0.86) | 0.88 (0.82, 0.94) | 0.99 (0.93, 1.06) | ~ |
|  | Pacific Islander | 1.07 (0.89, 1.27) | 0.94 (0.78, 1.13) | 1.27 (1.07, 1.50) | ~ |
| Distant | Black | 1.64 (1.57, 1.72) | 1.65 (1.57, 1.72) | 1.67 (1.60, 1.74) | 1.57 (1.50, 1.63) |
|  | Hispanic | 0.84 (0.79, 0.88) | 0.91 (0.86, 0.95) | 0.94 (0.89, 0.98) | 0.94 (0.90, 0.98) |
|  | American Indian | 0.87 (0.70, 1.08) | 1.05 (0.87, 1.26) | 1.33 (1.13, 1.55) | 1.31 (1.13, 1.51) |
|  | API | 0.82 (0.77, 0.88) | 0.86 (0.81, 0.92) | 0.87 (0.82, 0.92) | 0.83 (0.78, 0.87) |
|  | East Asian | 0.89 (0.82, 0.97) | 0.88 (0.81, 0.96) | 0.87 (0.80, 0.95) | ~ |
|  | South Asian | 0.33 (0.23, 0.47) | 0.38 (0.29, 0.50) | 0.50 (0.40, 0.63) | ~ |
|  | Southeast Asian | 0.76 (0.67, 0.85) | 0.87 (0.79, 0.96) | 0.95 (0.87, 1.05) | ~ |
|  | Pacific Islander | 1.01 (0.75, 1.30) | 1.73 (1.42, 2.08) | 1.66 (1.36, 2.02) | ~ |

Abbreviation: IRR, Incidence rate ratio (using White patients as the reference group); CI, Confidence Interval; API, Asian or Pacific Islander (aggregate), Early stage includes in-situ and localized tumors.

^a^ Early stage includes in-situ and localized tumors

**~** IRRs for API subgroups not available for the most recent time period (2015-2019)

Supplemental Table S2. Trends in stage-specific incidence and proportional stage distribution of Proximal adenocarcinomas by race/ethnicity from 2000 to 2019

|  |  | Age adjusted incidence rate (per 100,000) | | | | | Proportional stage distribution | | | | |
| --- | --- | --- | --- | --- | --- | --- | --- | --- | --- | --- | --- |
|  |  | 2000-2004 | 2005-2009 | 2010-2014 | 2015-2019 | APC | 2000-2004 | 2005-2009 | 2010-2014 | 2010-2014 | Δ |
| White | Early | 19.0 | 18.6 | 14.9 | 12.2 | -3.0* | 40.6% | 44.4% | 43.9% | 41.8% | 1.2* |
|  | Regional | 19.2 | 15.3 | 12.4 | 10.8 | -3.8* | 41.0% | 37.0% | 36.7% | 37.1% | -3.8* |
|  | Distant | 7.8 | 7.1 | 6.0 | 5.5 | -2.4* | 16.8% | 17.5% | 18.0% | 18.9% | +2.2* |
| Black | Early | 26.8 | 30.3 | 24.7 | 18.6 | -2.7* | 37.5% | 43.8% | 43.5% | 39.2% | +1.7 |
|  | Regional | 27.4 | 23.5 | 18.4 | 16.5 | -3.4* | 38.8% | 34.3% | 32.8% | 35.0% | -3.9* |
|  | Distant | 15.0 | 13.9 | 12.1 | 11.2 | -2.0* | 21.5% | 20.6% | 22.0% | 23.9% | 2.5* |
| Hispanic | Early | 12.8 | 13.3 | 12.8 | 10.6 | -1.3* | 37.7% | 40.5% | 43.2% | 38.6% | +0.9 |
|  | Regional | 14.2 | 12.9 | 10.5 | 10.8 | -1.9* | 42.6% | 39.4% | 36.3% | 40.4% | -2.1 |
|  | Distant | 5.9 | 6.0 | 5.4 | 4.9 | -1.4* | 18.2% | 18.8% | 18.8% | 18.7% | +0.5 |
| American Indian | Early | 16.6 | 15.7 | 16.9 | 14.0 | -1.1 | 42.2% | 41.3% | 41.9% | 41.1% | -1.1 |
|  | Regional | 15.2 | 14.3 | 16.3 | 12.2 | -1.5 | 39.2% | 37.0% | 39.5% | 35.9% | -3.3 |
|  | Distant | 6.0 | 7.6 | 6.5 | 6.9 | -0.2^ | 16.6% | 21.3% | 16.9% | 20.7% | +4.1 |
| API | Early | 10.7 | 11.8 | 10.5 | 7.8 | -2.1* | 36.2% | 43.0% | 44.0% | 39.4% | 3.2* |
|  | Regional | 13.3 | 10.3 | 8.9 | 8.0 | -3.3* | 45.1% | 37.5% | 37.8% | 40.7% | -4.4* |
|  | Distant | 5.1 | 4.9 | 3.9 | 3.3 | -3.0* | 17.5% | 18.4% | 16.6% | 17.1% | -0.4 |
| East Asian | Early | 12.2 | 12.2 | 10.8 | ~ | -0.9 | 38.3% | 43.0% | 43.3% | 40.3% | 2.1 |
|  | Regional | 13.8 | 11.0 | 9.1 | ~ | -4.0* | 43.6% | 38.7% | 38.4% | 39.4% | -4.2 |
|  | Distant | 5.5 | 4.8 | 3.9 | ~ | -3.2* | 17.0% | 17.2% | 16.7% | 18.4% | +1.4 |
| Southeast Asian | Early | 6.2 | 8.3 | 8.4 | ~ | 3.1 | 31.4% | 42.0% | 42.3% | 36.8% | +5.5* |
|  | Regional | 9.7 | 7.6 | 7.3 | ~ | -3.1* | 47.3% | 38.1% | 39.7% | 41.4% | -5.9* |
|  | Distant | 4.1 | 3.5 | 2.9 | ~ | -3.5* | 20.5% | 19.2% | 16.9% | 18.2% | -2.4 |
| South Asian | Early | 4.7 | 5.0 | 5.7 | ~ | ^b^ | 29.6% | 34.2% | 42.9% | 42.0% | +12.4 |
|  | Regional | 5.8 | 5.4 | 3.8 | ~ | ^b^ | 52.1% | 45.1% | 36.4% | 43.1% | -9.0 |
|  | Distant | 1.7 | 2.2 | 1.9 | ~ | ^b^ | 11.3% | 18.9% | 17.9% | 10.6% | -0.6 |
| Pacific Islander | Early | 13.2 | 13.6 | 14.0 | ~ | ^b^ | 40.7% | 41.7% | 43.4% | 35.1% | -5.6 |
|  | Regional | 14.2 | 6.7 | 11.0 | ~ | ^b^ | 42.5% | 25.7% | 37.4% | 41.9% | -0.6 |
|  | Distant | 5.0 | 8.6 | 4.6 | ~ | ^b^ | 15.0% | 28.5% | 19.3% | 21.5% | +6.4 |

APC, Average percent change; API, Asian or Pacific Islander (aggregate), Early stage includes in-situ and localized tumors

* p<0.05

Δ absolute value percent difference between 2000–2004 proportion value and 2015–2019 proportion value for each respective disease stage

^b^ Rates/Trends based on fewer than 5 cases not reported

~ IRs and IR trends for API subgroups not available for the most recent time period (2015-2019)

Supplemental table S3. Trends in stage-specific incidence and proportional stage distribution of Distal colon adenocarcinomas by race/ethnicity from 2000 to 2019

|  |  | Age adjusted incidence rate (per 100,000) | | | | | Proportional stage distribution | | | | |
| --- | --- | --- | --- | --- | --- | --- | --- | --- | --- | --- | --- |
|  |  | 2000-2004 | 2005-2009 | 2010-2014 | 2015-2019 | AAPC | 2000-2004 | 2005-2009 | 2010-2014 | 2010-2014 | Δ |
| White | Early | 18.5 | 14.5 | 11.1 | 9.1 | -4.7* | 51.6% | 50.8% | 48.7% | 44.5% | -7.1* |
|  | Regional | 11.2 | 8.9 | 6.9 | 6.7 | -3.6* | 31.1% | 31.2% | 30.5% | 32.6% | +1.6* |
|  | Distant | 5.6 | 4.7 | 4.3 | 4.0 | -2.1* | 15.6% | 16.4% | 19.1% | 20.0% | +4.3* |
| Black | Early | 19.7 | 17.8 | 13.5 | 10.1 | -4.5* | 45.8% | 46.1% | 44.5% | 40.7% | -5.1* |
|  | Regional | 13.9 | 12.1 | 9.2 | 7.9 | -3.8* | 31.7% | 32.1% | 30.4% | 31.5% | -0.3 |
|  | Distant | 8.6 | 7.5 | 6.7 | 6.1 | -2.3* | 20.0% | 19.9% | 22.8% | 24.3% | +4.3* |
| Hispanic | Early | 14.0 | 12.3 | 10.2 | 8.5 | -3.3* | 49.3% | 47.3% | 46.4% | 42.1% | -7.2* |
|  | Regional | 9.1 | 8.7 | 7.0 | 6.8 | -2.2* | 32.4% | 33.8% | 31.9% | 33.6% | +1.2 |
|  | Distant | 4.7 | 4.4 | 4.2 | 4.1 | -0.9* | 17.0% | 17.2% | 19.6% | 20.6% | +3.6* |
| American Indian | Early | 13.4 | 14.7 | 13.2 | 11.7 | -1.4 | 44.2% | 54.8% | 46.3% | 45.7% | +1.5 |
|  | Regional | 11.9 | 6.9 | 9.1 | 8.1 | -1.4 | 38.2% | 24.9% | 31.7% | 32.5% | -5.7 |
|  | Distant | 5.2 | 4.7 | 5.8 | 4.7 | b | 16.4% | 19.2% | 20.7% | 18.4% | +2.0 |
| API | Early | 16.5 | 14.8 | 12.7 | 10.2 | -3.1* | 46.9% | 46.1% | 46.1% | 41.9% | -5.1* |
|  | Regional | 12.7 | 11.5 | 9.3 | 8.8 | -2.5* | 36.3% | 35.8% | 34.1% | 35.9% | -0.4 |
|  | Distant | 5.4 | 5.2 | 4.8 | 4.6 | -0.9 | 15.5% | 16.4% | 17.9% | 18.8% | +3.4* |
| East Asian | Early | 18.2 | 15.8 | 12.6 | ~ | -3.6* | 46.7% | 46.1% | 45.6% | 40.0% | -6.7* |
|  | Regional | 14.8 | 13.1 | 10.0 | ~ | -3.6* | 37.5% | 37.3% | 35.3% | 39.1% | +1.7 |
|  | Distant | 5.7 | 5.1 | 4.6 | ~ | -1.8 | 14.4% | 14.7% | 16.8% | 18.0% | +3.6* |
| Southeast Asian | Early | 14.2 | 15.8 | 13.4 | ~ | -0.2 | 45.2% | 45.9% | 45.7% | 39.2% | -6.0* |
|  | Regional | 12.4 | 10.9 | 10.1 | ~ | -1.8 | 36.4% | 34.2% | 34.2% | 35.2% | -1.3 |
|  | Distant | 5.4 | 6.2 | 5.6 | ~ | -0.4 | 17.2% | 18.2% | 18.8% | 22.3% | +5.1* |
| South Asian | Early | 6.8 | 5.6 | 6.3 | ~ | ^b^ | 41.5% | 43.4% | 45.2% | 43.9% | +2.5 |
|  | Regional | 5.4 | 5.2 | 6.4 | ~ | ^b^ | 34.2% | 40.7% | 38.0% | 34.9% | +0.7 |
|  | Distant | 3.2 | 1.7 | 2.3 | ~ | ^b^ | 22.0% | 13.3% | 15.1% | 17.2% | -4.8 |
| Pacific Islander | Early | 24.8 | 19.1 | 14.0 | ~ | ^b^ | 56.6% | 41.0% | 43.3% | 42.3% | -14.3* |
|  | Regional | 15.2 | 13.2 | 9.7 | ~ | -4.3* | 30.9% | 33.1% | 27.3% | 32.8% | +2.0 |
|  | Distant | 5.5 | 10.9 | 10.8 | ~ | ^b^ | 11.8% | 24.7% | 28.3% | 20.9% | +9.1* |

* p<.05; APC, Average percent change; API, Asian or Pacific Islander (aggregate), Early stage includes in-situ and localized tumors

Δ absolute value percent difference between 2000–2004 proportion value and 2015–2019 proportion value for each respective disease stage

^b^ Trends based on fewer than 5 cases in a given year are not reported

~ IRs and IR trends for API subgroups not available for the most recent time period (2015-2019)

Supplemental Table S4. Trends in stage-specific incidence and proportional stage distribution of Rectal adenocarcinomas by race/ethnicity from 2000 to 2019

|  |  | Age adjusted incidence rate (per 100,000) | | | | | Proportional stage distribution | | | | |
| --- | --- | --- | --- | --- | --- | --- | --- | --- | --- | --- | --- |
|  |  | 2000-2004 | 2005-2009 | 2010-2014 | 2015-2019 | AAPC | 2000-2004 | 2005-2009 | 2010-2014 | 2010-2014 | Δ |
| White | Early | 18.3 | 14.8 | 11.6 | 9.7 | -4.2* | 49.5% | 47.1% | 41.7% | 37.1% | -12.4* |
|  | Regional | 12.0 | 10.5 | 10.3 | 10.2 | -1.1* | 32.6% | 33.6% | 37.2% | 38.7% | +6.2* |
|  | Distant | 5.4 | 5.2 | 5.1 | 5.3 | 0.1 | 14.6% | 16.6% | 18.5% | 20.6% | +6.0* |
| Black | Early | 13.8 | 13.3 | 10.4 | 7.7 | -3.9* | 42.1% | 43.2% | 38.9% | 34.0% | -8.1* |
|  | Regional | 10.6 | 10.0 | 9.2 | 8.0 | -1.9* | 32.4% | 32.9% | 34.6% | 35.7% | +3.3* |
|  | Distant | 6.5 | 6.0 | 5.9 | 5.7 | -0.5 | 20.2% | 19.9% | 22.7% | 26.0% | +5.8* |
| Hispanic | Early | 12.4 | 11.7 | 9.8 | 8.5 | -2.7* | 42.6% | 41.9% | 38.6% | 34.6% | -8.0* |
|  | Regional | 10.7 | 9.7 | 9.4 | 9.7 | -0.6* | 36.4% | 35.4% | 38.0% | 39.5% | +3.1* |
|  | Distant | 5.0 | 5.1 | 4.7 | 4.8 | -0.3 | 17.2% | 18.6% | 19.3% | 19.9% | +2.7* |
| American Indian | Early | 10.7 | 11.9 | 12.1 | 9.6 | -0.5 | 40.1% | 42.1% | 36.5% | 30.2% | -9.9* |
|  | Regional | 9.8 | 10.5 | 11.5 | 13.5 | 1.9 | 37.4% | 37.6% | 36.1% | 42.3% | +4.9 |
|  | Distant | 5.4 | 4.9 | 8.2 | 7.8 | b | 19.7% | 18.5% | 24.7% | 24.5% | +4.8 |
| API | Early | 14.8 | 12.8 | 11.1 | 8.5 | -3.6* | 44.5% | 43.1% | 40.8% | 35.2% | -9.4* |
|  | Regional | 12.0 | 11.0 | 10.0 | 10.1 | -1.2* | 36.6% | 37.5% | 37.1% | 41.3% | +4.7* |
|  | Distant | 5.4 | 4.8 | 4.6 | 4.6 | -0.9* | 16.5% | 16.5% | 17.4% | 18.8% | +2.3* |
| East Asian | Early | 15.1 | 12.9 | 10.9 | ~ | -3.7* | 43.5% | 42.3% | 40.9% | 35.3% | -8.2* |
|  | Regional | 13.1 | 11.7 | 9.2 | ~ | -3.4* | 37.6% | 38.5% | 37.3% | 42.1% | +4.5* |
|  | Distant | 5.7 | 4.9 | 4.1 | ~ | -2.7* | 16.5% | 16.4% | 16.6% | 17.4% | +0.9 |
| Southeast Asian | Early | 13.4 | 11.8 | 11.3 | ~ | -2.2* | 44.5% | 41.4% | 40.1% | 32.1% | -12.5* |
|  | Regional | 10.7 | 10.8 | 9.8 | ~ | -1.3 | 36.4% | 39.2% | 37.2% | 41.5% | +5.1* |
|  | Distant | 4.8 | 4.8 | 4.8 | ~ | -0.2 | 16.5% | 17.1% | 18.1% | 21.9% | +5.4* |
| South Asian | Early | 7.8 | 6.7 | 5.7 | ~ | -3.5* | 48.2% | 48.4% | 39.8% | 36.3% | -11.9 |
|  | Regional | 4.6 | 4.8 | 5.4 | ~ | b | 35.3% | 30.3% | 38.6% | 46.1% | +10.8 |
|  | Distant | 1.7 | 2.2 | 3.0 | ~ | b | 14.1% | 14.8% | 19.3% | 14.6% | +0.5 |
| Pacific Islander | Early | 16.4 | 13.9 | 13.6 | ~ | -2.0 | 40.2% | 41.1% | 32.9% | 34.7% | -5.5 |
|  | Regional | 14.5 | 11.6 | 14.4 | ~ | -0.4 | 37.8% | 35.4% | 38.5% | 38.3% | +0.5 |
|  | Distant | 8.2 | 9.0 | 8.5 | ~ | b | 21.3% | 22.2% | 22.5% | 24.8% | +3.5 |

APC, Average percent change; API, Asian or Pacific Islander (aggregate), Early stage includes in-situ and localized tumors

* p<.05

Δ absolute value percent difference between 2000–2004 proportion value and 2015–2019 proportion value for each respective disease stage

Rates/Trends based on fewer than 5 cases not reported

~ IRs and IR trends for API subgroups not available for the most recent time period (2015-2019)

Supplemental table S5. Five-year cumulative age-adjusted IRRs (compared with whites) of colorectal cancer by subsite, stage at diagnosis, and race/ethnicity

| Subsite/stage | Race/ethnicity | 2000-2004  IRR (95% CI) | 2005-2009  IRR (95% CI) | 2010-2014  IRR (95% CI) | 2015-2019  IRR (95% CI) |
| --- | --- | --- | --- | --- | --- |
| Proximal/Early | Black | **1.41 (1.34, 1.48)** | **1.63 (1.56, 1.71)** | **1.65 (1.58, 1.73)** | **1.53 (1.46, 1.61)** |
|  | Hispanic | **0.67 (0.63, 0.72)** | **0.72 (0.68, 0.76)** | **0.86 (0.82, 0.91)** | **0.87 (0.82, 0.91)** |
|  | American Indian | 0.87 (0.69, 1.08) | 0.85 (0.68, 1.04) | 1.13 (0.94, 1.36) | 1.16 (0.96, 1.38) |
|  | API | **0.56 (0.52, 0.61)** | **0.64 (0.60, 0.68)** | **0.70 (0.66, 0.75)** | **0.65 (0.60, 0.69)** |
|  | East Asian | **0.71 (0.64, 0.79)** | **0.73 (0.66, 0.80)** | **0.82 (0.74, 0.91)** | **~** |
|  | South Asian | **0.28 (0.17, 0.42)** | **0.30 (0.21, 0.40)** | **0.43 (0.33, 0.56)** | **~** |
|  | Southeast Asian | **0.36 (0.30, 0.43)** | **0.50 (0.43, 0.57)** | **0.64 (0.57, 0.73)** | **~** |
|  | Pacific Islander | 0.77 (0.54, 1.06) | 0.82 (0.60, 1.09) | 1.07 (0.81, 1.39) | **~** |
| Proximal/Regional | Black | **1.43 (1.36, 1.50)** | **1.54 (1.46, 1.62)** | **1.48 (1.41, 1.56)** | **1.53 (1.46, 1.61)** |
|  | Hispanic | **0.74 (0.69, 0.79)** | **0.84 (0.79, 0.89)** | **0.85 (0.80, 0.90)** | 1.01 (0.96, 1.06) |
|  | American Indian | **0.79 (0.62, 1.00)** | 0.94 (0.74, 1.16) | **1.31 (1.08, 1.58)** | 1.13 (0.93, 1.37) |
|  | API | **0.69 (0.64, 0.74)** | **0.67 (0.62, 0.72)** | **0.72 (0.67, 0.77)** | **0.75 (0.70, 0.80)** |
|  | East Asian | **0.80 (0.72, 0.88)** | **0.79 (0.72, 0.88)** | **0.84 (0.75, 0.93)** | **~** |
|  | South Asian | **0.34 (0.23, 0.48)** | **0.42 (0.30, 0.56)** | **0.35 (0.25, 0.48)** | **~** |
|  | Southeast Asian | **0.56 (0.49, 0.65)** | **0.55 (0.48, 0.63)** | **0.68 (0.59, 0.77)** | **~** |
|  | Pacific Islander | 0.82 (0.59, 1.12) | **0.48 (0.31, 0.72)** | 1.01 (0.73, 1.36) | **~** |
| Proximal/Distant | Black | **1.92 (1.79, 2.05)** | **1.95 (1.82, 2.08)** | **2.01 (1.88, 2.14)** | **2.02 (1.9, 2.16)** |
|  | Hispanic | **0.76 (0.69, 0.83)** | **0.84 (0.77, 0.92)** | **0.89 (0.82, 0.97)** | **0.89 (0.83, 0.96)** |
|  | American Indian | 0.77 (0.53, 1.10) | 1.07 (0.79, 1.42) | 1.08 (0.80, 1.43) | 1.25 (0.96, 1.61) |
|  | API | **0.66 (0.59, 0.74)** | **0.69 (0.62, 0.77)** | **0.65 (0.58, 0.72)** | **0.60 (0.55, 0.67)** |
|  | East Asian | **0.77 (0.65, 0.89)** | **0.74 (0.63, 0.86)** | **0.75 (0.63, 0.88)** | **~** |
|  | South Asian | **0.22 (0.09, 0.44)** | **0.34 (0.20, 0.53)** | **0.37 (0.23, 0.57)** | **~** |
|  | Southeast Asian | **0.57 (0.45, 0.7)** | **0.54 (0.44, 0.66)** | **0.55 (0.44, 0.67)** | **~** |
|  | Pacific Islander | 0.70 (0.38, 1.18) | 1.31 (0.90, 1.86) | 0.88 (0.53, 1.37) | **~** |
| Distal/Early | Black | **1.06 (1.00, 1.13)** | **1.22 (1.16, 1.29)** | **1.22 (1.15, 1.29)** | **1.11 (1.04, 1.18)** |
|  | Hispanic | **0.76 (0.71, 0.80)** | **0.85 (0.80, 0.90)** | **0.92 (0.87, 0.97)** | **0.93 (0.88, 0.98)** |
|  | American Indian | **0.72 (0.56, 0.91)** | 1.01 (0.81, 1.24) | 1.19 (0.97, 1.45) | **1.28 (1.04, 1.55)** |
|  | API | **0.89 (0.83, 0.95)** | 1.02 (0.96, 1.08) | **1.15 (1.08, 1.22)** | **1.12 (1.06, 1.19)** |
|  | East Asian | 0.97 (0.89, 1.05) | 1.06 (0.97, 1.15) | **1.16 (1.06, 1.27)** | **~** |
|  | South Asian | **0.36 (0.25, 0.50)** | **0.38 (0.28, 0.51)** | **0.59 (0.46, 0.75)** | **~** |
|  | Southeast Asian | **0.75 (0.67, 0.84)** | 1.06 (0.96, 1.16) | **1.23 (1.11, 1.35)** | **~** |
|  | Pacific Islander | **1.32 (1.03, 1.66)** | 1.28 (0.99, 1.62) | 1.28 (0.98, 1.66) | **~** |
| Distal/Regional | Black | **1.24 (1.16, 1.33)** | **1.36 (1.27, 1.46)** | **1.34 (1.24, 1.43)** | **1.19 (1.11, 1.28)** |
|  | Hispanic | **0.82 (0.76, 0.88)** | 0.97 (0.91, 1.04) | 1.01 (0.94, 1.08) | 1.02 (0.96, 1.09) |
|  | American Indian | 1.07 (0.81, 1.38) | 0.77 (0.56, 1.05) | **1.31 (1.02, 1.66)** | 1.22 (0.96, 1.54) |
|  | API | **1.14 (1.06, 1.23)** | **1.29 (1.20, 1.38)** | **1.35 (1.26, 1.45)** | **1.32 (1.24, 1.41)** |
|  | East Asian | **1.23 (1.12, 1.36)** | **1.41 (1.28, 1.55)** | **1.40 (1.26, 1.55)** | **~** |
|  | South Asian | **0.46 (0.31, 0.65)** | **0.58 (0.42, 0.78)** | 0.91 (0.71, 1.16) | **~** |
|  | Southeast Asian | 1.04 (0.91, 1.17) | **1.18 (1.04, 1.32)** | **1.43 (1.28, 1.6)** | **~** |
|  | Pacific Islander | 1.27 (0.93, 1.71) | **1.42 (1.04, 1.89)** | 1.37 (0.98, 1.86) | **~** |
| Distal/Distant | Black | **1.54 (1.41, 1.69)** | **1.61 (1.47, 1.75)** | **1.57 (1.45, 1.71)** | **1.50 (1.38, 1.63)** |
|  | Hispanic | **0.84 (0.75, 0.93)** | 0.95 (0.86, 1.05) | 0.99 (0.9, 1.08) | 1.02 (0.94, 1.10) |
|  | American Indian | 0.93 (0.60, 1.36) | 1.00 (0.68, 1.41) | 1.36 (0.99, 1.83) | 1.15 (0.83, 1.56) |
|  | API | 0.96 (0.85, 1.07) | 1.11 (1.00, 1.23) | **1.13 (1.02, 1.24)** | **1.14 (1.04, 1.25)** |
|  | East Asian | 0.96 (0.82, 1.12) | 1.03 (0.88, 1.20) | 1.08 (0.93, 1.25) | **~** |
|  | South Asian | **0.52 (0.30, 0.83)** | **0.36 (0.19, 0.61)** | **0.55 (0.35, 0.81)** | **~** |
|  | Southeast Asian | 0.91 (0.75, 1.1) | **1.25 (1.07, 1.46)** | **1.32 (1.14, 1.53)** | **~** |
|  | Pacific Islander | 0.92 (0.52, 1.5) | **2.20 (1.56, 3.01)** | **2.52 (1.82, 3.40)** | **~** |
| Rectum/Early | Black | **0.75 (0.70, 0.81)** | **0.90 (0.84, 0.95)** | **0.90 (0.84, 0.96)** | **0.80 (0.74, 0.86)** |
|  | Hispanic | **0.68 (0.63, 0.72)** | **0.79 (0.74, 0.84)** | **0.85 (0.80, 0.90)** | **0.87 (0.82, 0.92)** |
|  | American Indian | **0.58 (0.44, 0.76)** | 0.80 (0.62, 1.01) | 1.05 (0.84, 1.29) | 0.99 (0.79, 1.22) |
|  | API | **0.81 (0.76, 0.87)** | **0.86 (0.81, 0.92)** | 0.96 (0.9, 1.02) | **0.88 (0.82, 0.94)** |
|  | East Asian | **0.86 (0.78, 0.94)** | 0.93 (0.84, 1.02) | 1.02 (0.93, 1.12) | **~** |
|  | South Asian | **0.45 (0.32, 0.60)** | **0.47 (0.34, 0.61)** | **0.53 (0.41, 0.68)** | **~** |
|  | Southeast Asian | **0.76 (0.68, 0.86)** | **0.85 (0.75, 0.95)** | 1.06 (0.95, 1.17) | **~** |
|  | Pacific Islander | 0.97 (0.72, 1.28) | 0.99 (0.74, 1.32) | 1.31 (0.99, 1.69) | **~** |
| Rectum/Regional | Black | **0.88 (0.81, 0.95)** | 0.94 (0.88, 1.02) | **0.90 (0.83, 0.96)** | **0.78 (0.73, 0.84)** |
|  | Hispanic | **0.89 (0.83, 0.96)** | **0.92 (0.86, 0.98)** | **0.92 (0.87, 0.98)** | 0.95 (0.90, 1.01) |
|  | American Indian | 0.81 (0.61, 1.06) | 0.99 (0.76, 1.27) | 1.12 (0.9, 1.37) | **1.33 (1.10, 1.59)** |
|  | API | 1.01 (0.93, 1.08) | 1.04 (0.97, 1.11) | 0.97 (0.91, 1.04) | 1.01 (0.94, 1.06) |
|  | East Asian | **1.11 (1.01, 1.23)** | **1.16 (1.05, 1.28)** | 0.96 (0.86, 1.06) | **~** |
|  | South Asian | **0.40 (0.26, 0.58)** | **0.46 (0.32, 0.63)** | **0.57 (0.44, 0.74)** | **~** |
|  | Southeast Asian | 0.91 (0.79, 1.03) | 1.07 (0.95, 1.20) | 1.02 (0.91, 1.13) | **~** |
|  | Pacific Islander | 1.23 (0.89, 1.65) | 1.15 (0.83, 1.55) | **1.52 (1.15, 1.95)** | **~** |
| Rectum/Distant | Black | **1.21 (1.1, 1.34)** | **1.15 (1.04, 1.27)** | **1.17 (1.07, 1.27)** | 1.08 (0.99, 1.17) |
|  | Hispanic | 0.93 (0.84, 1.03) | 0.98 (0.89, 1.07) | 0.93 (0.86, 1.01) | **0.90 (0.83, 0.97)** |
|  | American Indian | 1.02 (0.66, 1.45) | 0.94 (0.64, 1.34) | **1.62 (1.24, 2.08)** | **1.46 (1.14, 1.85)** |
|  | API | 1.01 (0.90, 1.12) | 0.93 (0.83, 1.03) | 0.91 (0.82, 1.02) | **0.86 (0.78, 0.94)** |
|  | East Asian | 1.08 (0.92, 1.26) | 0.98 (0.84, 1.14) | 0.86 (0.74, 1.01) | **~** |
|  | South Asian | **0.31 (0.15, 0.58)** | **0.43 (0.25, 0.69)** | **0.63 (0.43, 0.90)** | **~** |
|  | Southeast Asian | 0.91 (0.74, 1.10) | 0.97 (0.81, 1.15) | 1.02 (0.86, 1.19) | **~** |
|  | Pacific Islander | 1.56 (0.99, 2.33) | **1.79 (1.23, 2.53)** | **1.78 (1.25, 2.47)** | **~** |

IRR, Incidence rate ratio (using White patients as the reference group); CI, Confidence Interval; API, Asian or Pacific Islander (aggregate), Early stage includes in-situ and localized tumors. Bolded values denote statistical significance (p <.05)

**~** IRRs for API subgroups not available for the most recent time period (2015-2019)

**Supplemental Figure S1.** Age-adjusted incidence trends of early, regional and distant CRC by race/ethnicity from 2000-2019, from Surveillance, Epidemiology, and End Results (SEER) 17 registries (November 2021 Submission). API, Asian/Pacific Islander (aggregate of all subgroups)

**Supplemental Figure S2.** Age-adjusted incidence trends of early stage, regional stage and distant stage CRC among API subgroups from 2000-2014, from Surveillance, Epidemiology, and End Results (SEER) Detailed Asian/Pacific Islander subgroup incidence and population dataset (November 2016 Submission). Note: Incidence data for API subgroups not available for the most recent time period (2015-2019)
